# Small RNA Sequencing Identifies PIWI-Interacting RNAs Deregulated in Glioblastoma—piR-9491 and piR-12488 Reduce Tumor Cell Colonies *In Vitro*

**DOI:** 10.3389/fonc.2021.707017

**Published:** 2021-08-13

**Authors:** Michael Bartos, Frantisek Siegl, Alena Kopkova, Lenka Radova, Jan Oppelt, Marek Vecera, Tomas Kazda, Radim Jancalek, Michal Hendrych, Marketa Hermanova, Petra Kasparova, Zuzana Pleskacova, Vaclav Vybihal, Pavel Fadrus, Martin Smrcka, Radek Lakomy, Radim Lipina, Tomas Cesak, Ondrej Slaby, Jiri Sana

**Affiliations:** ^1^Department of Neurosurgery, University Hospital Hradec Kralove, Hradec Kralove, Czechia; ^2^Central European Institute of Technology, Masaryk University, Brno, Czechia; ^3^Department of Radiation Oncology, Masaryk Memorial Cancer Institute and Medical Faculty, Masaryk University, Brno, Czechia; ^4^Department of Neurosurgery, St. Anne’s University Hospital and Medical Faculty, Masaryk University, Brno, Czechia; ^5^1st Department of Pathology, St. Anne’s University Hospital and Medical Faculty, Masaryk University, Brno, Czechia; ^6^The Fingerland Department of Pathology, University Hospital Hradec Kralove, Hradec Kralove, Czechia; ^7^Department of Oncology and Radiotherapy, University Hospital Hradec Kralove, Hradec Kralove, Czechia; ^8^Department of Neurosurgery, University Hospital Brno, Brno, Czechia; ^9^Department of Comprehensive Cancer Care, Masaryk Memorial Cancer Institute, Brno, Czechia; ^10^Department of Neurosurgery, University Hospital Ostrava, Ostrava, Czechia; ^11^Department of Biology, Faculty of Medicine, Masaryk University, Brno, Czechia; ^12^Department of Pathology, University Hospital Brno, Brno, Czechia

**Keywords:** glioblastoma, PIWI-interacting RNA, piRNA, diagnosis, prognosis

## Abstract

Glioblastoma (GBM) is the most frequently occurring primary malignant brain tumor of astrocytic origin. To change poor prognosis, it is necessary to deeply understand the molecular mechanisms of gliomagenesis and identify new potential biomarkers and therapeutic targets. PIWI-interacting RNAs (piRNAs) help in maintaining genome stability, and their deregulation has already been observed in many tumors. Recent studies suggest that these molecules could also play an important role in the glioma biology. To determine GBM-associated piRNAs, we performed small RNA sequencing analysis in the discovery set of 19 GBM and 11 non-tumor brain samples followed by TaqMan qRT-PCR analyses in the independent set of 77 GBM and 23 non-tumor patients. Obtained data were subsequently bioinformatically analyzed. Small RNA sequencing revealed 58 significantly deregulated piRNA molecules in GBM samples in comparison with non-tumor brain tissues. Deregulation of piR-1849, piR-9491, piR-12487, and piR-12488 was successfully confirmed in the independent groups of patients and controls (all *p* < 0.0001), and piR-9491 and piR-12488 reduced GBM cells’ ability to form colonies *in vitro*. In addition, piR-23231 was significantly associated with the overall survival of the GBM patients treated with Stupp regimen (*p* = 0.007). Our results suggest that piRNAs could be a novel promising diagnostic and prognostic biomarker in GBM potentially playing important roles in gliomagenesis.

## Introduction

Glioblastoma (GBM) is the most aggressive of the glial tumors and is related to a very poor prognosis with a median survival between 14 and 15 months (1, 2). GBM originates from astrocytes, which have a largely supportive function in the nervous system (1). Based on the genetic pathway of their formation, two types of GBM can be distinguished. Primary GBM arises *de novo* and secondary GBM develops as a continuation of low-grade glioma. Primary GBM has a higher prevalence in elderly people, whereas secondary GBM is more often associated with younger age (1, 3). The most significant prognostic factors are age at the time of diagnosis, size and localization of the tumor, Karnofsky performance status, and the genetic makeup of the tumor (4). Currently, the gold standard of therapy in GBM consists of maximal surgical resection followed by concomitant radiotherapy and chemotherapy with temozolomide (TMZ) (5). In the past two decades, regardless of improvements in general oncological care through intensive research, the recommended treatment in GBM remains largely unchanged and the overall survival (OS) of GBM patients has not significantly improved. Therefore, a great effort is devoted to gaining a better understanding of GBM biology and the research of novel predictive biomarkers as well as potential therapeutic targets. One of the very promising areas of research in potential biomarkers and molecules likely involved in the molecular pathology of GBM are PIWI-interacting RNAs (piRNA). PiRNAs present one of the subclasses of short non-coding RNAs, 24–32 nucleotides in length, which act as regulators of active transposons by induction of heterochromatinization in transposon loci or by direct cleavage of complementary transposon transcripts in piRISC complexes by the associated PIWI proteins [PIWIL1 (HIWI), PIWIL2 (HILI), PIWIL3 (HIWI3), and PIWIL4 (HIWI2)] (6, 7). PiRNA expression was primarily reported in germline cells where they play a crucial role in stem-cell maintenance and cell renewal regulation (7). Although originally thought to be germline-specific, piRNA have subsequently been associated with numerous functions in somatic cells ranging from epigenetic regulation, genome rearrangement, somatic cell development, to gene and protein regulatory functions (6). The aberrant expression of piRNA and PIWI has also recently been described in many cancers including their regulatory roles in tumor biology (8). Nevertheless, to this day, only a few studies have been published on their role in GBM (8, 9).

In this study, we identified a set of piRNAs that are likely closely associated with GBM in comparison to non-tumor brain tissue. These piRNAs may thus play an important role in the pathogenesis of GBM and could present a potential therapeutic target in GBM. Finally, we demonstrated the prognostic potential of piRNAs by showing the ability of piR-23231 to predict OS in GBM patients treated with Stupp regimen independently of the other prognostic markers.

## Materials and Methods

### Patient Cohort and Primary Cell Cultures

The retrospective multi-institutional (University Hospital Brno, University Hospital Hradec Kralove, University Hospital Ostrava, and St Anne’s University Hospital Brno) cohort study included 96 patients with histopathologically confirmed primary GBM and 34 non-tumor controls. All patients enrolled in this study gave consent and the study was approved by the local ethics committees. The clinical and pathological characteristics of GBM patients are summarized in **Table 1**. Non-tumor control brain tissues were obtained *via* therapeutic resections in patients with intractable epilepsy—only brain tissue lacking evidence of dysplastic changes from the non-dominant temporal or frontal lobe was used. Forty-one GBM patients underwent adjuvant concomitant chemoradiotherapy (CHRT) according to the Stupp protocol. In summary, 60 Gy of fractionated radiotherapy was administered to the primary site of the tumor, followed by 42 cycles of TMZ chemotherapy. A subset of patients were further indicated to adjuvant TMZ in monotherapy.

**Table 1:** Clinical, histopathological, and molecular characteristics of patients included in the study.

Variable	All patients		Patients with concomitant chemoradiotherapy	
	Explorative phase	Validation phase	Explorative phase	Validation phase
Number of patients	19	77	11	30
Age (years; median, range)	64 (42–75)	64 (30–80)	60 (42–71)	61 (33–74)
Gender (men)	11 (58%)	41 (53%)	6 (55%)	19 (63%)
IDH 1/2 mutation	0 (0%)	6 (8%)	0 (0%)	2 (7%)
MGMT methylation	5 (26%)	22 (29%)	3 (27%)	9 (30%)
Concomitant RT with TMZ	11 (58%)	36 (47%)	11 (100%)	30 (100%)

Moreover, six primary GBM cell cultures were derived from fresh tumor tissue samples obtained from GBM patients who underwent surgical resection at the Department of Neurosurgery of the University Hospital Brno. The fresh tissue sample was enzymatically dissociated with TrypLE (ThermoFisher Scientific) for 20 min at 37°C with agitation or using the Papain Dissociation System (Worthington) according to the manufacturer’s instructions. Single-cell suspensions were seeded into 25 cm^2^ tissue culture flasks (Techno Plastic Products AG) and cultured in Dulbecco’s modified essential medium supplemented with 10% FBS, 1% Glutamax (both ThermoFisher Scientific), 100 U/ml penicillin and 100 μg/ml streptomycin, 1mM sodium pyruvate and 1% non-essential amino acids (all GE Healthcare). After 1–3 weeks, adherent cells, which covered more than 2/3 of the culture flask in DMEM, were passaged using Trypsin–EDTA solution (Sigma-Aldrich). For the subsequent analyses of selected piRNAs’ expression levels, early passage cultures were used (10).

### Tissue Sample Preparation and Nucleic Acid Extraction

All tissue samples were frozen and stored at −80°C in RNA stabilization solution—RNAlater (ThermoFisher Scientific, cat. n. AM7021). Fresh-frozen tissues were homogenized with ceramic beads and total RNA enriched with small RNA fractions was isolated using a mirVana miRNA Isolation Kit with phenol (Invitrogen). The concentration of extracted RNA was measured using UV spectrophotometry (NanoDrop 2000 Spectrophotometer, ThermoFisher Scientific) and the integrity of the obtained RNA was assessed *via* electrophoresis on 1% agarose gel. The RNA integrity number (RIN) was assessed with capillary electrophoresis (2200 Tape Station, Agilent).

### piRNA Expression Profile Analysis With Next-Generation Sequencing

cDNA libraries for next-generation sequencing were prepared using the CleanTag Small RNA Library Preparation Kit (TriLink Biotechnologies, cat. n. L3206). Concentrations of cDNA libraries were assessed by fluorimetry (Qubit 2.0, ThermoFisher Scientific), and the integrity of libraries was measured by capillary electrophoresis (2200 TapeStation, Agilent). Subsequently, selection of fragments corresponding to a specific length of sncRNA with added adaptors was performed on cDNA libraries using gel electrophoresis (Pippin Prep, Sage Science). Selected cDNA library fragments underwent next-generation sequencing [NextSeq 500/550 High Output v2 kit (75 cycles)] (Illumina, cat. n. FC-404-2005) on a NextSeq 500 Sequencing System (Illumina). Raw fastq reads were quality checked with FastQC (v0.11.5) and Kraken (v15-065). 3′end adapters were trimmed with Cutadapt (v1.15). The trimmed sequences were size filtered for expected piRNA sizes (24–32 bp), and low-quality ends (Phred < 10) were removed with Cutadapt (v1.15). Statistics from all the preprocessing steps were summarized with MultiQC (v1.4).

Known contaminants (rRNA, tRNA, snoRNA, snRNA, YRNA, and miRNA—stemloop; Ensembl 91) were removed from the preprocessed reads by mapping (end-to-end) the reads to their sequences with Bowtie (v1.2.1.1) with a maximum of 1 mismatch. The preprocessed and cleaned reads were mapped (end-to-end) to human piRNA sequences downloaded from the piRBase database (v1.0) using Bowtie (v1.2.1.1) with a maximum of two mismatches. Raw Bowtie output was converted to SAM format using in-house Perl script and further processed with Samtools (v1.6), Picard (2.8.2), and cgat (v0.3.2). FeatureCounts (v1.5.0) were used to summarize the piRNA counts (minimal overlap of 24 bp). The multimapped reads were equally divided to the mapped references as fractions. Differential expression was calculated in R (v3.4.3) with package DESeq2 (v1.16.1). The criterion of adjusted *p*-value < 0.05 for differentially expressed piRNAs was considered alone, without any restrictions on logFC. The limma procedure, which does not require any other restrictions, was applied and Benjamini-Hochberg correction was then performed to adjust for multiple comparisons to control for false discovery rate. Only piRNAs with |logFC| > 0.5 were identified as differentially expressed.

### Quantification of piRNAs by qRT-PCR

For the synthesis of cDNA from RNA enriched by a fraction of small RNAs, 6.66 ng of RNA was used. For reverse transcription, a TaqMan MicroRNA Reverse Transcription Kit (Applied Biosystems, cat. n. 4366596) was used. A LightCycler 480 Instrument II (Roche) together with a TaqMan MicroRNA Assay protocol (ThermoFisher Scientific) were used for qRT-PCR. The threshold cycle data were determined using the default threshold settings of the machine. All of the performed PCR reactions were made in duplicate, followed by calculation of the average Ct and SD values. For the evaluation of the results, the relative piRNA expression level was calculated by the 2^−ΔCT^ method. ΔCTs were calculated according to the following formula: ΔCT = CT(piRNA of interest) – CT[(CtmiR-103 + CtU6)/2]. As reference molecules, miR-103 and U6 were chosen based on the current literature. GraphPad Prism 8 software (San Diego, CA, USA) was used for the following statistical evaluation including Mann–Whitney non-parametric tests, ROC analysis, and Kaplan–Meier analysis, together with JMP 14.3.0 (Cary, NC, USA), which was used for combined ROC analysis and univariable analysis. As a threshold of significance, a *p*-value = 0.05 was selected. ROC curve analysis was used to determine the area under the curve (AUC) including 95% confidence interval (CI) values, and cutoff points were set to achieve the highest levels of sensitivity and specificity. In the case of ROC focused on piR-23231 distinguishing the ability between better and poorer prognosis samples, the criterion of division of samples into two groups was set to survival longer or shorter than 15 months. For Kaplan–Meier survival analysis, the log-rank test was used to determine statistical significance. The piR-23231 relative expression value dividing GBM samples/patients into lower and higher piR-23231 expression groups has been determined to be 29.27 using ROC analysis.

### Stable Cell Lines and Reagents

For the *in vitro* part of the study, GBM cell line U-251 MG was chosen and maintained in DMEM supplemented with 10% FBS, 1 mM sodium pyruvate, 2 mM glutamine, 100 units/ml of penicillin, and 100 µg/ml of streptomycin. piRNA mimetics with 3’methylated end nucleotides were purchased from IDT—piRNA mimetics piR-hsa-9491—5′UGAAUCUGACAACAGAGGCUUACGACCCCUUmA3′; piR-hsa-12488—5′ CAGAGUGUAGCUUAACACAAAGCACCCAACUmG3′; also, piRNA-like non-targeting RNA sequence was used as scrambled oligonucleotide control 5′ACGCCACGUCUUAUAUUAACACAACGGUGAGmC3′. For *in vitro* assays, reverse transfection was used using LipofectAMINE RNAiMAX transfection reagent (Invitrogen) according to the manufacturer’s protocol. Upregulation of specific piRNA levels was confirmed by qRT-PCR.

Furthermore, commercial stable normal human astrocytes (N7805100, ThermoFisher Scientific) were used for analysis of selected piRNAs’ expression levels. These cells are maintained in DMEM supplemented with 10% FBS and 1% N2 supplement (all ThermoFisher Scientific).

### MTT Cell Viability Assay

Cells were transfected with piRNA mimetics and scrambled oligonucleotides; also, LipofectAMINE RNAiMAX without RNA oligonucleotides was used as MOCK negative control. MTT (3-(4,5-dimethylthiazol-2-yl)-2,5-diphenyltetrazolium bromide) was added to the cells after 24 h from transfection and left to metabolize for 2 h. Then, the medium with MTT was aspirated and formazan crystals were dissolved in DMSO (dimethyl sulfoxide). Absorbance was measured using the FLUOstar Omega reader. The method was performed in five independent repetitions in different times; each repetition was performed in hexaplicate.

### Colony Formation Assay

One hundred fifty cells per well were seeded and transfected with selected oligonucleotides; also, MOCK was used as control. Cells were left to grow for 10 days and then stained with crystal violet and analyzed using ImageJ v1.8.0 software. Experiments were done in hexaplicate, and five repeated experiments were performed. Colony numbers were analyzed in GraphPad Prism 8 using *t*-test.

## Results

### Global Expression Analysis of piRNAs in GBM Tissue Samples and Non-Tumor Brain Control

**Figure 1:**
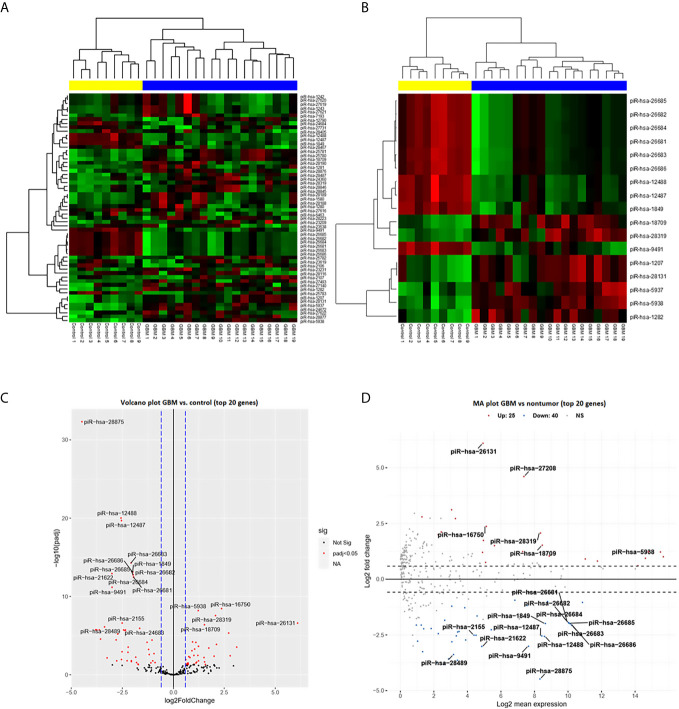
PiRNA expression profiling based on next-generation sequencing data. **(A)** Clustering analysis with heatmap based on the expression of 58 identified piRNAs meeting the criteria of more than 50 reads in at least 50% of samples. Samples were divided into a tumor group (blue) and a control group (yellow) with 100% sensitivity and 100% specificity. Thirty-five piRNAs were upregulated (red) in glioblastoma and 23 were downregulated (green) in glioblastoma. **(B)** Clustering analysis of 17 selected differentially expressed piRNAs (adj. *p* < 0.0001) in glioblastoma compared to non-tumor controls. **(C)** Volcano plot of the dependency of the log2 adjusted *p*-value on log2 Fold Change of all analyzed (*n* = 58) piRNAs. Red dots represent significantly dysregulated piRNAs (adj. *p* < 0.05); 20 genes with the highest results are described by their names. **(D)** Volcano plot of dependency of the Log2 fold change on Log2 mean expression of all analyzed (*n* = 58) piRNAs. Red dots present significantly dysregulated piRNAs (adj. *p* < 0.05); 20 genes with the highest results are described by their names.

From a set of 595 detected piRNA molecules, 58 molecules accomplished criteria of detection in at least 50% samples with more than 50 reads in GBM tumor tissues (*n* = 19) and non-tumor samples (*n* = 11) [median of average expressions = 10.2565; median of log fold change (logFC) = 0.3873] (**Figure 1**). Following statistical evaluation using limma approach identified 15 significantly downregulated piRNAs in GBM samples, whereas 23 of piRNA molecules were significantly upregulated in GBM (*p*_adjust_ < 0.05) (**Table 2**). After the tightening of criteria (*p* < 0.0001), 17 piRNA molecules showed significant dysregulation between tumors and controls.

**Table 2:** List of the significantly dysregulated PIWI-interacting RNAs in GBM patients compared to non-tumor controls based on next-generation sequencing data.

Upregulated piRNAs in GBM				Downregulated piRNAs in GBM			
piRNA	logFC	AveExpr	adj. *p*-value	piRNA	logFC	AveExpr	adj. *p*-value
**piR-hsa-5938**	**1.429**	**17.409**	**<0.001**	**piR-hsa-12488**	**−2.355**	**10.116**	**<0.001**
piR-hsa-28319	1.851	9.843	<0.001	**piR-hsa-12487**	**−2.335**	**9.971**	**<0.001**
piR-hsa-18709	1.700	10.275	<0.001	**piR-hsa-9491**	**−3.159**	**8.670**	**<0.001**
piR-hsa-5937	1.123	16.535	<0.001	**piR-hsa-1849**	**−1.875**	**10.348**	**<0.001**
piR-hsa-1207	1.342	16.538	<0.001	piR-hsa-26683	−1.953	11.898	<0.001
piR-hsa-28131	1.341	16.538	<0.001	piR-hsa-26686	−1.883	11.842	<0.001
piR-hsa-1282	1.102	12.993	<0.001	piR-hsa-26685	−1.848	11.778	<0.001
piR-hsa-25781	1.189	10.880	<0.001	piR-hsa-26682	−1.844	11.765	<0.001
piR-hsa-28877	1.147	17.55	<0.001	piR-hsa-26684	−1.826	11.748	<0.001
piR-hsa-25780	1.099	10.725	0.001	piR-hsa-26681	−1.825	11.742	<0.001
piR-hsa-24672	0.846	16.164	0.001	piR-hsa-24684	−1.17	9.249	0.001
piR-hsa-28876	0.881	9.845	0.006	piR-hsa-25783	−0.904	12.762	0.002
piR-hsa-27493	0.856	13.67	0.007	piR-hsa-28467	−0.949	10.640	0.007
piR-hsa-28487	0.821	10.189	0.015	piR-hsa-27616	−0.807	8.783	0.01
piR-hsa-1281	0.787	10.073	0.019	piR-hsa-27731	−0.718	9.427	0.052
piR-hsa-1580	0.742	8.714	0.019				
piR-hsa-28189	0.742	8.714	0.019				
piR-hsa-25782	0.614	11.677	0.025				
piR-hsa-23209	1.114	8.802	0.028				
piR-hsa-27622	0.724	15.945	0.025				
piR-hsa-27140	0.774	13.97	0.028				
piR-hsa-7193	0.706	9.222	0.03				
piR-hsa-28188	0.669	8.315	0.04				

Five piRNAs in bold were selected for further validation by qRT-PCR. log Fold Change = logFC, average expression = AveExpr, adjusted *p*-value = adj. *p*-value

### Validation of the Expression of Selected piRNAs in the Independent Cohort of the GBM Patient Tissue Samples Against Non-Tumor Controls and Primary GBM Cell Lines Against Normal Human Astrocytes

**Figure 2:**
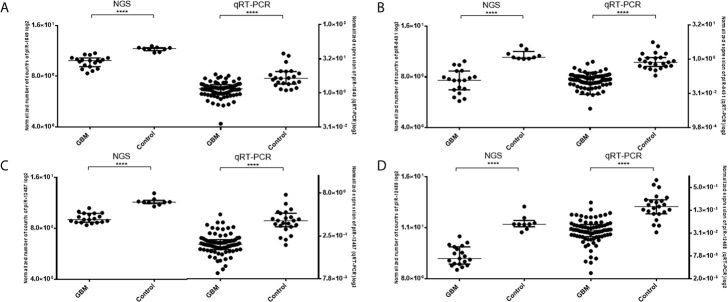
Results of Mann–Whitney analysis of independent validation of selected piRNAs and comparison between obtained results from next-generation sequencing and qRT-PCR. **(A)** piR-1849; **(B)** piR-9491; **(C)** piR-12487; **(D)** piR-12488. ****p < 0.0001.

**Figure 3:**
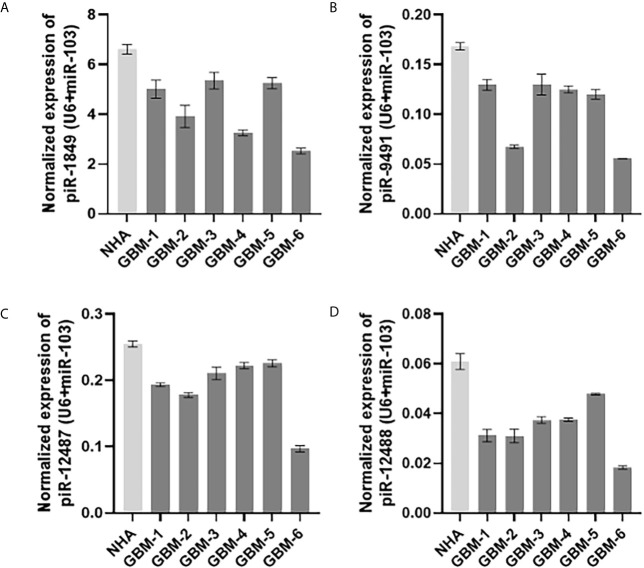
Expression levels of **(A)** piR-1849; **(B)** piR-9491; **(C)** piR-12487; **(D)** piR-12488 in normal human astrocytes (NHA) and primary glioblastoma cell lines analyzed by qRT-PCR.

**Figure 4:**
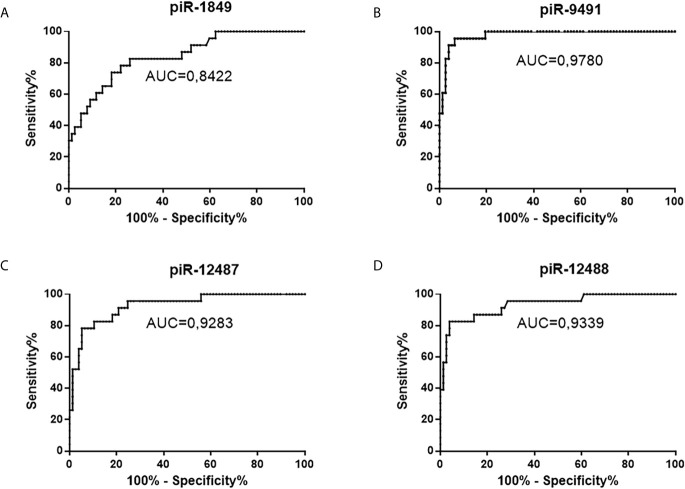
ROC analysis of all successfully validated piRNA molecules and their distinguishing ability between tumors and non-tumor controls. **(A)** piR-1849, AUC 0.8422, sensitivity 82.6%, specificity 74%; **(B)** piR-9491 AUC 0.978, sensitivity 100%, specificity 80.5%; **(C)** piR-12487 AUC 0.9283, sensitivity 91.3%, specificity 79.2%; **(D)** piR-12488 AUC 0.9339, sensitivity 87%, specificity 85.7%.

Five piRNAs (piR-hsa-1849, piR-hsa-5938, piR-hsa-9491, piR-hsa-12487, and piR-hsa-12488) selected based on NGS data as the five most significantly dysregulated piRNAs in GBMs were validated on an independent cohort of 77 GBM samples and 23 non-tumor controls. The qRT-PCR analysis using TaqMan stem-loop assays followed by Mann–Whitney analysis confirmed the results from NGS in four piRNAs (piR-1849, piR-9491, piR-12487, and piR-12488) (**Figure 2**). The same results were observed also when comparing primary GBM cell lines and normal human astrocytes (NHA) (**Figure 3**). ROC analyses that differentiate which piRNA/piRNAs have the best distinguishing effect between GBM tissue and non-tumor tissue reveal that the piR-9491 alone shows the best specificity and sensitivity in identifying GBM samples from non-tumor samples (AUC = 0.978) (**Figure 4**); specificity and sensitivity in both other standalone piRNAs and other piRNA combinations were lower.

### Tumor Tissue Expression of piR-23231 Is Associated With OS of GBM Patients

**Figure 5:**
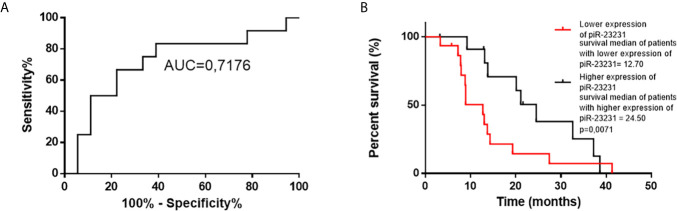
ROC and Kaplan–Meier analysis of piR-23231 on validation cohort of patients. **(A)** ROC analysis to determine the distinguishing ability of piR-23231 between patients with survival shorter and longer than 15 months. **(B)** Kaplan–Meier analysis of lower levels of piR-23231 against higher levels (*p* = 0.0071).

Cox regression analysis of OS of GBM patients and global piRNA expression profiles has shown associations with piR-28876, piR-23231, and piR-18709 (*p* < 0.1). Subsequent ROC analysis on the independent cohort of GBM patients who underwent completed Stupp protocol (radiotherapy 60 Gy, 42 cycles of TMZ, and possible adjuvant therapy with TMZ) confirmed that piR-23231 has been able to distinguish patients surviving less and more than 15 months with 75% sensitivity and 66.67% specificity (AUC = 0.7176; **Figure 5A**). Significant association of piR-23231 expression and OS has also been confirmed by Kaplan–Meier analysis (*p* = 0.0071; log-rank test). Specifically, lower expression of piR-23231 has been significantly associated with poorer survival (**Figure 5B**). Finally, univariate Cox regression analysis has confirmed the association of piR-23231 with survival in this examined cohort of GBM patients (*p* = 0.02).

### piR-hsa-9491 and piR-hsa-12488 Reduce the Ability to Form Colonies *In Vitro*

**Figure 6:**
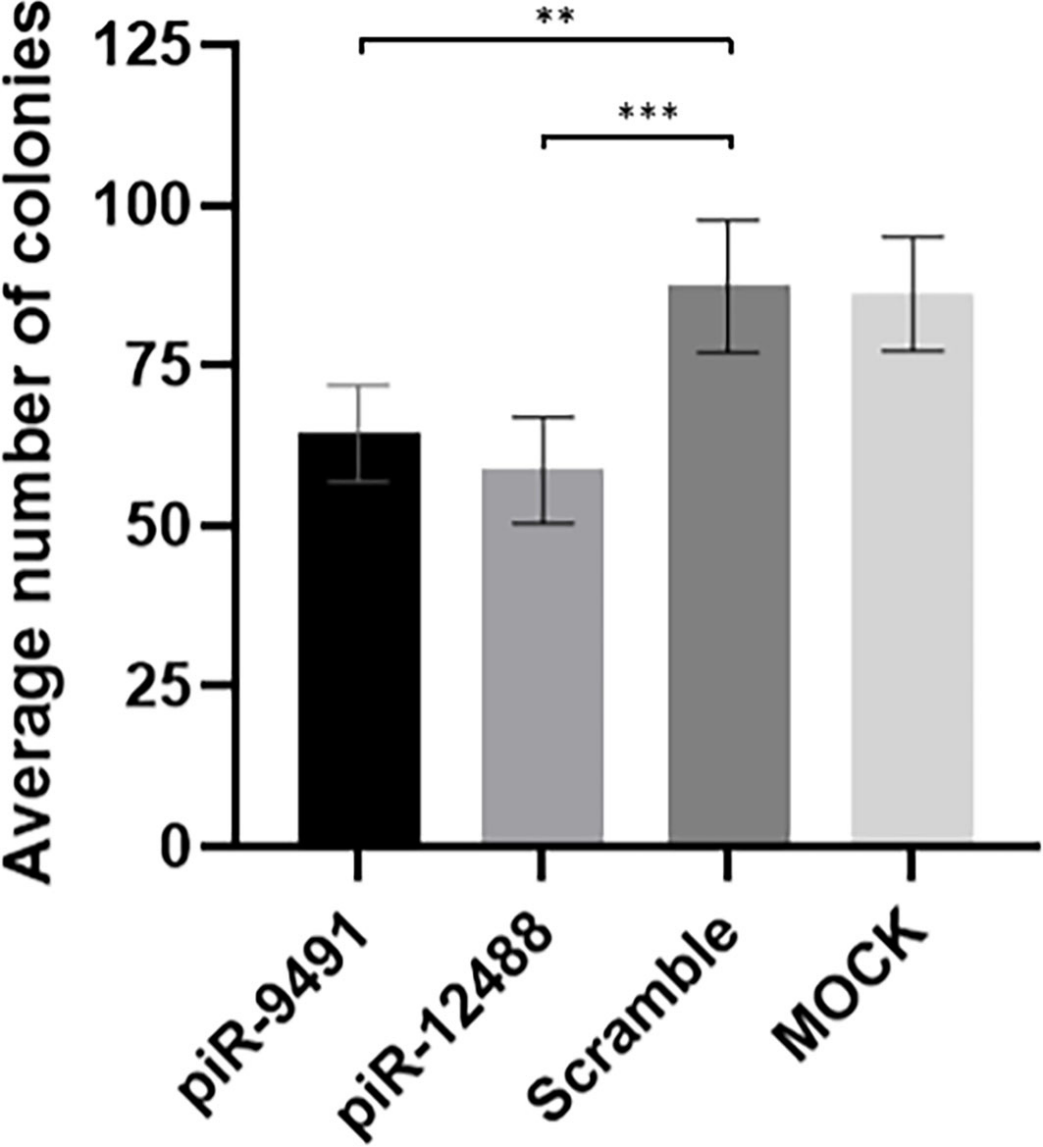
Colony formation analysis results for hsa-piR-9491 and hsa-piR-12488 compared to scrambled oligonucleotide and MOCK negative control. Increased levels of piR-9491 and piR-12488 led to decreased number of colonies compared to scrambled oligonucleotide and U251 MG cells treated with transfection reagent without oligonucleotide (MOCK). **p < 0.01, ***p < 0.001.

We performed *in vitro* transient transfection of piR-hsa-9491 and piR-has-12488 mimics in U-251 MG cell lines to investigate the effect of piR-hsa-9491 and piR-has-12488 levels on GBM cell viability and the ability to form colonies. Colony formation assays showed that both piR-hsa-9491 and piR-has-12488 significantly reduced the ability to form colonies in examined GBM cell line when compared with both control scrambled oligonucleotide and MOCK control (**Figure 6**). MTT viability assay did not show any significant results for selected piRNAs.

## Discussion

Despite improvements in oncological care, as well as refinements in neurosurgery and radiodiagnostics, the prognosis of GBM patients remains poor, and in addition, they often suffer from a severely diminished quality of life. This is mostly due to the aggressive and infiltrative character of the disease as well as its cellular and molecular heterogeneity. Brain tissue infiltration by tumor cells exceeds the resection borders; thus, recurrence relatively early into treatment is common. Even compared to other cancers, GBM has a low OS with a median of 14–15 months from diagnosis (1, 11). Treatment of patients with GBM remains an immense challenge; therefore, there is a great need for a better understanding of the molecular background of the disease, its origin, and development. Also, identification of new potential diagnostic and prognostic biomarkers, usable for better patient care management and the prediction of the course of the disease, and novel therapeutic targets could be greatly influential in the future GBM treatment (5). Due to the considerable heterogeneity prevailing between individual GBMs, new molecular markers for better categorization of individual tumors are also in high demand (12).

Dysregulation of short non-coding RNAs was described as a crucial action in the development of most tumors. Moreover, sncRNAs are involved in the vast majority of cellular processes; thus, disruption of their regulation is a crucial step in cancerogenesis. A subgroup of sncRNAs—piRNAs, as well as their associated PIWI proteins, were described as aberrantly expressed in many cancers, including GBM. Their dysregulation is often associated with poorer prognosis, which suggests their possible functional role in cancer biogenesis and therefore they present promising diagnostic or prognostic biomarkers, as well as potential therapeutic targets (13). Most of the tumors arise because of epigenetic changes, accumulation of mutations and genome destabilization. The piRNA/PIWI pathway is one of the key regulators in genome stability maintenance. Together, specific piRNA and PIWI proteins form a piRISC complex to control the activity of transposons, including their translocation within the genome. Disruption of the transcriptional and post-transcriptional regulatory role of piRNA molecules could possibly lead to the upregulation of transposons and their uncontrolled incorporation within the genome, leading to increased genome destabilization (14). Moreover, specific piRNAs could be responsible for the regulation of the expression of specific genes, involved in cancer development, growth, or maintenance (15–17). piRNAs with specific protein interactions were also described, extending the possible scoop of action of these molecules (18). Overall, the piRNA/PIWI pathway could play a significant role in cancerogenesis or therapy response of the tumors as significant dysregulation of piRNA molecules has been described in several studies and specific piRNAs as potential biomarkers or therapeutic targets have been proposed (19).

However, studies involving GBM or at least malignant gliomas are rarely seen; therefore, we decided to fill this gap. The aim of this study was to identify piRNAs associated with gliomagenesis, which could be potentially used for the diagnostics or prognosis determination. One of the first studies exploring piRNAs in the context of GBM was done by Jacobs et al., describing approximately 350 piRNA molecules in both tumor and stromal tissue using array-based piRNA expression profiling; among these, piR-8041 (piR-11270 in piRBase, DQ580941) was downregulated, and its upregulation in *in vivo* experiments showed remarkable tumor suppressive effects (9). Nevertheless, limitations of this study are a low number of patients in the piRNA profiling phase; only seven tumors and seven controls were included. Furthermore, the study lacks the validation phase with the independent cohort of patients. In our study, piR-8041 was not found in piRNAs significantly dysregulated in GBM compared to non-tumor controls, as it did not meet the criterion of minimum samples in which it was detected (50 reads at least in 50% samples). This finding could be caused by markedly wider sample size as well as different methodology used for the analysis. Going through glioma studies, Leng et al. described piR-20280 (DQ590027) as an important player in the piR-20280/MIR17HG/miR-153/FOXR2 pathway, playing a crucial role in the regulation of the permeability of glioma-conditioned normal brain–blood barrier (15). Liu et al. described piR-30188 as part of the PIWIL3/OIP5-AS/miR-367-3p/CEBPA pathway, which is downregulated in gliomas, and its overexpression together with overexpression of PIWIL3 and miR-367-3p lead to inhibition of glioma cell progression (16). Shen et al. described piR-23387 (DQ593109) as a molecule with an impact on the permeability of the blood–tumor barrier (17). However, those studies aimed different biological processes; specifically, they are not designed for the observation of molecules potentially involved in GBM development or progression of the disease; rather, they focused on the interesting topic of the changes of permeability of blood–brain barrier mediated by the presence of gliomas. This is the basis for the differences between our studies as the authors, mentioned above, use appropriate cell models instead of GBM tissue and different methodology. Despite the different aims of the studies, material, and methodology used, we were able to identify molecules from Shen et al. (piR-DQ593109) and Leng et al. (piR-DQ590027) in our dataset; unfortunately, they did not pass the criterion of at least 50 reads in 50% of the samples, which is completely understandable under the given study conditions and differences.

In the discovery phase of our study, we analyzed 58 piRNAs, selected by strict criteria (50 reads at least in 50% of samples). From this group, we found 23 significantly upregulated piRNAs in GBM in comparison to non-tumor controls and 15 downregulated molecules. From the group of 38 dysregulated piRNAs, several of them were already described as molecules related to cancer. Hashim et al. described piR-28877 (DQ598677) as upregulated, and piR-28467 (DQ598252) and piR-27616 (DQ597341) as downregulated in breast cancer, which corresponds with our results in GBM (20). Also, Huang et al. performed global profiling of piRNAs in breast cancer to find piR-27493 (DQ597218) and piR-7193 (DQ576872) as upregulated, which correspond with our findings in GBM (21). The same tendencies of dysregulation of abovementioned piRNAs possibly suggest cellular roles that could be conserved across different cell types. On the other hand, some of the piRNAs [piR-26682 (DQ596466), piR-26684 (DQ596468)] from the chrM:12206-12237:+ region were also described in a study from Chu et al. On the contrary, those piRNAs were described as upregulated in bladder cancer in contrast to our observation in GBM, suggesting the possible tissue-specific roles of those piRNAs (22). Interestingly, piRNAs from the same region [piR-26681 (DQ596465), piR-26683 (DQ596467), piR-26685 (DQ596469), piR-26686 (DQ596470)] are found to be downregulated in GBM, which may suggest a potential tumor suppressive role of this genomic region. piR-823 (piR-1282, DQ571031) is a well-known molecule described in many cancers with variable expression among different tumors. In multiple myeloma, it is upregulated and connected to *de novo* methylation and angiogenesis (23). However, in gastric cancers, piR-823 is significantly downregulated, contributing to tumor growth, and its increase suppresses tumor growth *in vivo* (24). In the context of GBM, piR-823 was found to be upregulated, corresponding to a case of multiple myeloma, suggesting possible similarities with the role of piR-823 in multiple myeloma. Our finding is also another evidence of the tissue-specific role of piR-823 in various tumors. Interestingly, piR-1849, which is one of the molecules we were interested in, was earlier identified as the molecule that is cargoed into the extracellular vesicles that are released from the GBM cells and could be a potentially promising biomarker of the disease (25).

In the explorative phase, piR-1849 (DQ571526), piR-5983 (DQ575705), piR-9491 (DQ579193), piR-12487 (DQ582264), and piR-12488 (DQ582265) (all adj. *p*-value < 0.0001) were selected for the validation on the independent cohort of 77 tumor samples and 23 non-tumor controls. We successfully validated piR-1849, piR-9491, piR-12487, and piR-12488 as significantly downregulated molecules in GBM. Subsequently, we performed ROC analyses to identify analytical characteristics of validated piRNAs in distinguishing ability between tumors and non-tumors. It should be mentioned that all selected piRNAs have strong distinguishing abilities alone. However, of all possible combinations of those four piRNAs and the same piRNAs standalone, piR-9491 showed the highest specificity, sensitivity, and AUC.

In search of piRNAs possibly linked to prognosis, we examined obtained data trying to find an association between piRNA expression patterns and OS of GBM patients. In order to increase the validity of our results, we selected only patients who had undergone concomitant chemoradiotherapy in the extent of the Stupp protocol in this part of the study. Kaplan–Meier analyses showed that only piR-23231 (DQ592953) was significantly connected with prognosis (*p* = 0.0071; log rank test) in GBM patients, and its lower level was associated with a worse prognosis. This coincides with our previous NGS analysis, where piR-23231 was significantly downregulated in GBM and possibly contributing to GBM behavior and poorer survival, which have to be confirmed by appropriate *in vitro* and *in vivo* analyses. Also, Cox regression analyses were performed to distinguish the effect of piR-23231 as a potential prognostic biomarker.

In addition to previously mentioned works, it must be noted that piRNA role in cancer is rather controversial due to the findings of Tosar et al. (26). Based on this study, most of the annotated piRNAs, including some piRNAs described in cancer or other pathophysiological conditions, could be considered as cellular waste or RNA degradation products, originating from rRNA, tRNA, and other RNA species. The main issue is that the large bulk of annotated piRNAs does not meet the main features of this group of molecules, as they lack 1U or 10A. On the other hand, based on the articles mentioned above, it is clear that even molecules lacking 1U can affect cellular qualities and behavior; in fact, two best-described piRNAs—hsa-piR-651 and hsa-piR-823—are lacking 1U. Rather than sequence qualities of each individual piRNA, it would be more convincing to determine whether the particular piRNA binds to PIWI proteins or is able to alter cellular properties or behavior in response to functional analyses.

To answer the previously mentioned issues, functional analyses of selected piRNAs were carried out in U251 MG cells. piRNA expression levels were increased using transient transfection methodology; results were confirmed by qRT-PCR. Analyses showed that higher levels of piR-has-9491 and piR-has-12488 led to the reduced GBM cells’ ability to form colonies. Therefore, we assume that these two piRNAs can alter cellular properties and, thus, they are functional molecules rather than degradation fragments of other RNA molecules. However, their specific role in cell biology and ability to bind PIWI proteins must be determined in future studies. Notwithstanding this fact, molecules analyzed in this study provide promising potential GBM biomarkers that could be used for the diagnosis assessment and prognosis determination, if future studies confirm their role in the GBM biology.

## Conclusions

This study presents the largest study so far focused on the global piRNA profiling in GBM tissue samples and non-tumor brain tissue specimens. We identified a set of piRNAs significantly deregulated in GBM from which piR-9491 and piR-12488 were able to reduce the ability to form tumor cell colonies *in vitro.* These two piRNAs seem to be interesting molecules for other investigations, mainly as therapeutic targets in GBM. Finally, expression levels of piR-23231 were associated with patients’ OS, suggesting that some piRNAs could also present prognostic biomarkers in GBM. Based on the results, identified piRNAs could be part of the machinery involved in the pathogenesis of gliomas, which needs to be examined in future studies.

## Data Availability Statement

The datasets presented in this study can be found in online repositories. The names of the repository/repositories and accession number(s) can be found below: NCBI SRA; PRJNA747758.

## Ethics Statement

The studies involving human participants were reviewed and approved by Local ethics committees at: Masaryk University, Brno; University Hospital Brno, Brno; University Hospital Hradec Kralove, Hradec Kralove. The patients/participants provided their written informed consent to participate in this study.

## Author Contributions

The proposed study was undertaken at several clinical and academic workplaces. All authors contributed to data acquisition, analyses, and manuscript preparation. Conceptualization: MB, OS, and JS. Data curation: LR, JO, VV, and PF. Formal analysis: FS, LR, and JO. Funding acquisition: OS and JS. Investigation: FS, TK, and MiH. Methodology: AK and MV. Project administration: TK and RLa. Resources: MB, RJ, VV, PF, MS, and RLi. Clinical Data: MaH, PK, ZP, and RLa. Supervision: MV, RJ, MaH, MS, RLi, TC, and JS. Validation: AK and MV. Visualization: FS and JS. Writing—original draft: FS and JS. Writing—review and editing: MB and OS. All authors contributed to the article and approved the submitted version.

## Funding

This work was financially supported by the Ministry of Health, Czech Republic grant no. NV19-03-00501, conceptual development of research organizations (MOU, 00209805; and FNBr, 65269705); Ministry of Education, Youth and Sports, Czech Republic under the project CEITEC 2020 (LQ1601); and European Regional Development Fund-Project BBMRI-CZ: Biobank network—a versatile platform for the research of the etiopathogenesis of diseases, No. EF16 013/0001674.

## Conflict of Interest

The authors declare that the research was conducted in the absence of any commercial or financial relationships that could be construed as a potential conflict of interest.

## Publisher’s Note

All claims expressed in this article are solely those of the authors and do not necessarily represent those of their affiliated organizations, or those of the publisher, the editors and the reviewers. Any product that may be evaluated in this article, or claim that may be made by its manufacturer, is not guaranteed or endorsed by the publisher.
